# Persimmon-induced excessive anticoagulation in a patient with mechanical heart valves receiving warfarin therapy: A case report

**DOI:** 10.1097/MD.0000000000047861

**Published:** 2026-03-06

**Authors:** JunSeok Kim, SongAm Lee, HyeongJu Moon, WooSurng Lee

**Affiliations:** a Department of Thoracic and Cardiovascular Surgery, School of Medicine, Konkuk University, Konkuk University Seoul Hospital, Seoul, Republic of Korea; b Department of Thoracic and Cardiovascular Surgery, School of Medicine, Konkuk University, Konkuk University Chungju Hospital, Chungju-si, Chungbuk, Republic of Korea.

**Keywords:** anticoagulant, Coumadin, drug interaction, persimmons, vitamin K antagonist, warfarin

## Abstract

**Rationale:**

Warfarin is a widely used vitamin K antagonist requiring strict international normalized ratio monitoring due to its narrow therapeutic window. While the interaction between warfarin and vitamin K-rich foods is well known, limited data exist regarding the anticoagulant effects of persimmons, a fruit rich in various bioactive compounds.

**Patient concerns:**

A 51-year-old woman with a history of double mechanical valve replacement presented for routine follow-up. She reported no symptoms or changes in medication but had consumed unripe persimmons (“Dangam”) daily for 2 weeks prior to a sudden elevation in coagulation parameters.

**Diagnoses:**

Routine laboratory testing revealed elevated prothrombin time and activated partial thromboplastin time without any signs of bleeding or other clinical abnormalities. The timing of the abnormal results suggested a possible food–drug interaction.

**Interventions:**

The patient was advised to discontinue persimmon intake immediately. Warfarin dosage was reduced from 4.25 to 3.5 mg without administering vitamin K supplementation. Coagulation parameters were monitored during this adjustment period.

**Outcomes:**

Within 2 weeks, prothrombin time and activated partial thromboplastin time values returned to baseline. The patient remained stable on a slightly adjusted warfarin dose (3.25–3.9 mg), with no further abnormalities over the subsequent 3 years of follow-up. Over 9.5 years, she completed 63 visits with consistent international normalized ratio control.

**Lessons:**

This case highlights a potential food–drug interaction between persimmons and warfarin leading to excessive anticoagulation. The mechanism may involve more than vitamin K content alone, warranting further investigation. Physicians should consider dietary habits, including local or seasonal foods, in warfarin-treated patients presenting with unexpected coagulation changes.

## 1. Introduction

Warfarin is a widely used oral anticoagulant (OAC) with a narrow therapeutic window and therefore requires careful dose adjustment and regular monitoring to balance thromboembolic prevention against bleeding risk. As a vitamin K antagonist (VKA), warfarin inhibits the production of vitamin K-dependent clotting factors, rendering its anticoagulant effect particularly sensitive to dietary intake and drug–drug interactions. Despite the increasing global use of direct oral anticoagulants (DOACs), warfarin remains the standard and recommended anticoagulant therapy for patients with mechanical heart valves, in whom DOACs are not recommended. In this population, maintaining stable anticoagulation is essential, and even modest changes in dietary exposure may lead to clinically meaningful fluctuations in coagulation parameters. Food–drug interactions are a well-recognized challenge in warfarin management, particularly with foods rich in vitamin K or other bioactive constituents that may influence coagulation pathways. Persimmons (*Diospyros kaki*), commonly consumed in East Asia, contain a range of nutrients and phytochemicals that could plausibly affect anticoagulation; however, clinical evidence describing persimmon–warfarin interactions remains limited. Here, we report a case of excessive anticoagulation temporally associated with persimmon consumption in a patient with mechanical heart valves receiving long-term warfarin therapy.^[1-4]^

## 2. Case presentation

**Figure 1.**
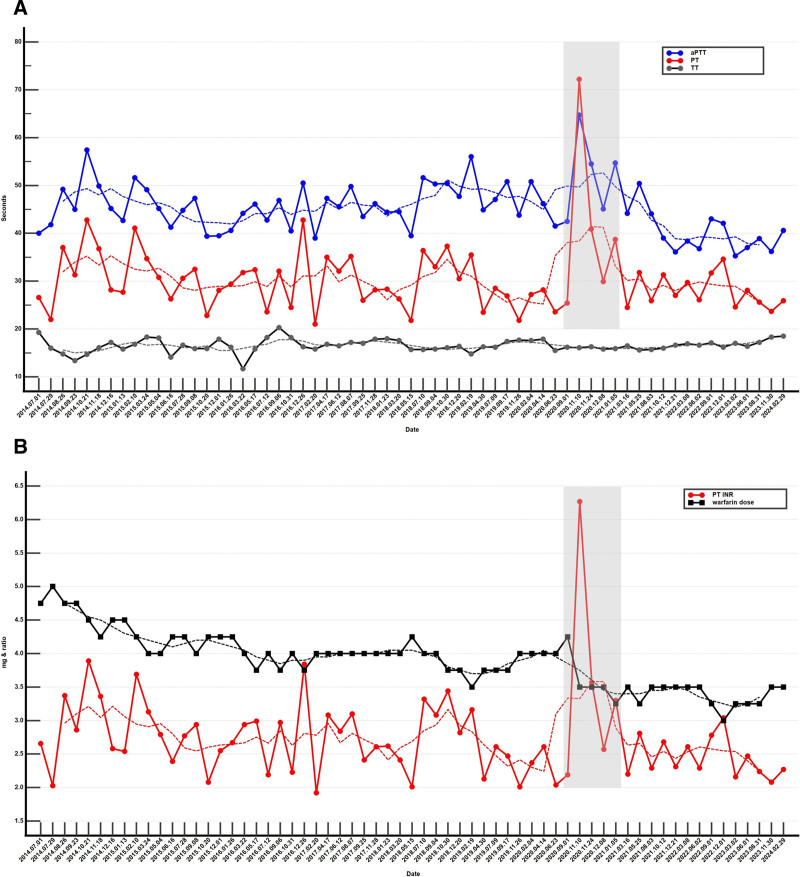
(A) Consecutive coagulation tests, including aPTT (normal range: 21.1–33.5), PT (normal range: 9.1–12.3), and TT (normal range: 14.3–19.2), were conducted on a 51-year-old woman who has been undergoing warfarin anticoagulation therapy since her cardiac surgery, including mechanical replacements of the mitral and aortic valves, from July 1, 2014, to the present. A gray box highlights a period of hypercoagulation due to persimmon consumption. Each dotted line represents the trend line of the respective coagulation tests (aPTT, PT, TT). (B) The figure shows the consecutive PT INR, (normal range: 0.88–1.19) levels and warfarin doses. A gray box highlights a period of hypercoagulation attributed to persimmon consumption, while each dotted line represents the trend line of the respective the INR levels and warfarin doses. aPTT = activated partial thromboplastin time, INR = international normalized ratio, PT = prothrombin time, TT = thrombin time.

**Figure 2.**
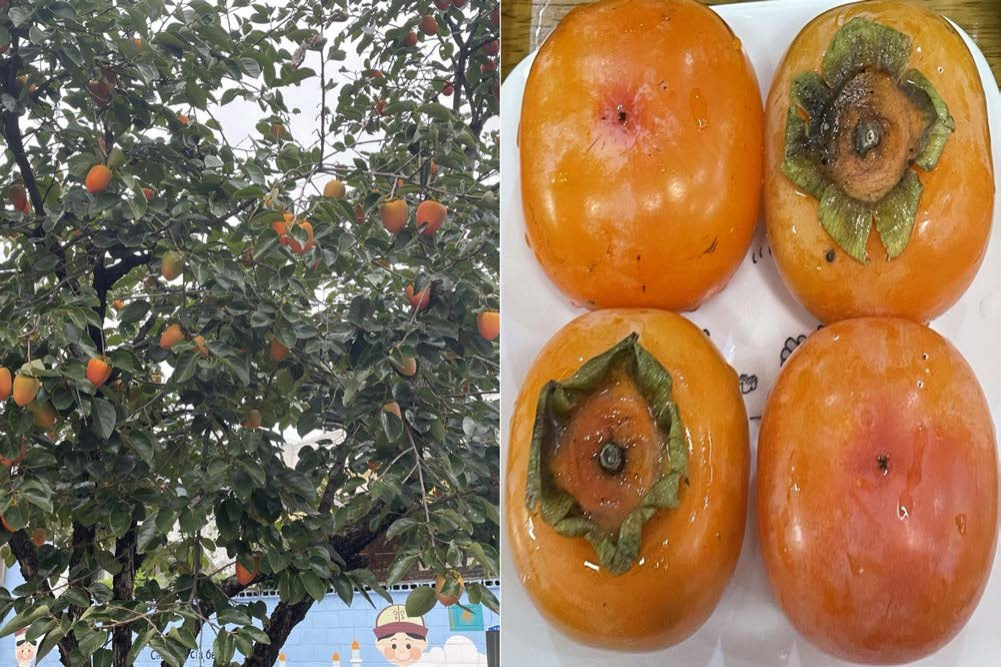
In Korea, persimmons (*Diospyros kaki*) are traditionally classified into 3 main types based on their ripeness and preparation method: “Dangam,” firm and unripe persimmons eaten fresh; “Hongsi,” fully ripened and soft persimmons, also known culturally as “Mulleong-gam”; and “Gotgam,” persimmons that are peeled and dried before consumption. The image shows a real persimmon tree (*D kaki*) growing along the wall of the hospital where I work, which produces fruit annually in the fall. Persimmons harvested directly from this tree without any postharvest treatment are referred to as “Dangam” in Korean. When these persimmons are allowed to ripen naturally at room temperature, they are called “Hongsi.” The subsequent image illustrates a “Hongsi,” showcasing a persimmon that has undergone natural ripening postharvest.

A 51-year-old woman has been followed at our Department of Thoracic and Cardiovascular Surgery for long-term warfarin anticoagulation since July 2014. She underwent mitral and aortic valve replacement at a University Hospital in Seoul, the capital of the Republic of Korea, in 1988, followed by redo double valve replacement at a University Hospital in Busan, the second-largest city in the Republic of Korea, in 2008. Mechanical prosthetic valves were implanted, including a St. Jude Medical 27-mm valve in the mitral position and a 17-mm valve in the aortic position. After the second operation, she remained clinically stable and continued warfarin therapy without complications. The patient was referred to our institution on July 1, 2014, due to relocation. This retrospective case report was approved by the Institutional Review Board of Konkuk University Chungju Hospital (IRB No. KUCH 2024-02-003), and the requirement for written informed consent was waived. Initial coagulation studies demonstrated therapeutic anticoagulation, including a prothrombin time (PT) of 26.6 seconds, an activated partial thromboplastin time (aPTT) of 40.0 seconds, an international normalized ratio (INR) of 2.66, and a thrombin time (TT) of 19.3 seconds. Warfarin was prescribed at 3.5 mg daily, targeting an INR range of 2.75 to 3.0, consistent with anticoagulation recommendations for patients with double mechanical heart valves. The patient received standardized education regarding warfarin use and potential dietary interactions and maintained a consistent diet. She was followed regularly with serial coagulation testing. The patient demonstrated good adherence to warfarin therapy and dietary counseling, with no evidence of noncompliance. In November 2020, routine laboratory evaluation revealed a sudden elevation in coagulation parameters: PT 72.2 seconds, aPTT 64.7 seconds, INR 6.27, and TT 16.1 seconds (Table 1; Figs. 1 and 2). At that time, the patient was asymptomatic, and vital signs and physical examination findings were unremarkable. There were no changes in concomitant medications or overall dietary patterns. However, dietary history revealed consumption of approximately 3 servings/day of firm, unripe persimmons (“Dangam”) for 2 consecutive weeks prior to the abnormal laboratory findings. Given the temporal association between persimmon intake and excessive anticoagulation, persimmon consumption was discontinued. Warfarin therapy was not interrupted; instead, the daily dose was temporarily reduced from 4.25 to 3.50 mg for 2 weeks. No vitamin K supplementation was administered during this period. After cessation of persimmon intake, coagulation parameters returned to the therapeutic range, and no further abnormalities were observed. The patient has since remained clinically stable while receiving approximately 3.25 mg of warfarin daily, without bleeding or thromboembolic complications. Over 9 years and 8 months of follow-up (July 2014 to February 2024), she completed 63 outpatient visits at our institution. During this period, the mean PT, aPTT, INR, and TT values were 30.5, 45.2, 2.7, and 16.5 seconds, respectively, and the mean daily warfarin dose was 3.9 mg (Table 1; Figs. 1 and 2).

**Table 1:** The table presents representative coagulation profiles and corresponding warfarin dosages monitored throughout the outpatient follow-up period.

Parameters	Reference range	2014	2014	2015	2016	2017	2018	2019	2020	2020	2020	2020	2020	2021	2022	2023	2024	Average
		07-01	07-29	01-13	01-26	02-20	01-23	02-19	02-04	09-01	**11-10**	11-24	12-08	01-05	03-08	03-02	02-29	
PT	[9.1–12.3] s	26.6	22.0	27.7	29.4	21.0	28.3	35.5	27.2	25.4	**72.2** *	40.9	29.9	38.7	29.7	24.6	25.9	30.47
aPTT	[21.1–33.5] s	40.0	41.8	42.7	40.6	39.0	44.4	56.0	50.8	42.5	**64.7** *	54.5	45.1	54.7	38.4	35.3	40.6	45.17
INR	[0.88–1.19]	2.66	2.03	2.54	2.67	1.92	2.62	3.16	2.37	2.19	**6.27** *	3.57	2.57	3.30	2.61	2.16	2.27	2.74
TT	[14.3–19.2] s	19.3	16.0	15.8	16.2	15.8	18.0	14.8	17.6	16.2	**16.1**	16.3	15.8	15.9	16.9	17.0	18.5	16.55
Warfarin dose	mg	4.75	5.00	4.50	4.25	4.00	4.00	3.50	4.00	4.25	**3.50**	3.50	3.50	3.25	3.50	3.25	3.50	3.90

aPTT = activated partial thromboplastin time, INR = international normalized ratio, PT = prothrombin time, TT = thrombin time.

* An asterisk and bold text indicate a period of hypercoagulation due to persimmon consumption.

## 3. Discussion

Given the high variability in drug response related to pharmacokinetics and pharmacodynamics, together with its narrow therapeutic window, strict monitoring of prothrombin clotting time – standardized as the INR – and subsequent dose adjustments are essential for VKAs such as warfarin. The target therapeutic INR range is typically 2 to 3 in most clinical settings, as subtherapeutic and supratherapeutic values are associated with increased risks of thromboembolism and hemorrhage, respectively. Accordingly, strict adherence to prescribed warfarin therapy to maintain the INR within the therapeutic range is of critical clinical importance. Persimmons, originating from China and Japan, are widely consumed for their sweet flavor and soft texture when ripe, with representative varieties including the Japanese persimmon (*D kaki*) and the American persimmon (*Diospyros virginiana*). In addition to their palatability, persimmons are rich in vitamins A and C, dietary fiber, potassium, and other essential nutrients. Notably, persimmons contribute to dietary vitamin K intake, a key factor in coagulation and bone metabolism, which is also abundant in leafy green vegetables, broccoli, Brussels sprouts, and certain fruits. Consequently, the consumption of vitamin K-rich persimmons has the potential to interfere with the anticoagulant effect of warfarin, a VKA, particularly in patients with mechanical heart valves.^[1-4]^ The development of warfarin originated from investigations into hemorrhagic disease in cattle exposed to spoiled sweet clover, which led to the identification of dicoumarol and the subsequent synthesis of coumarin-derived anticoagulants. In 1948, the Wisconsin Alumni Research Foundation patented a more potent compound, naming it “warfarin,” derived from Wisconsin Alumni Research Foundation and coumarin. Although initially introduced as a rodenticide, its anticoagulant properties soon prompted clinical application for the prevention and treatment of thromboembolic disorders in the early 1950s, and warfarin sodium later became widely used under the brand-name Coumadin. Despite major advances in anticoagulation therapy with the introduction of newer agents, warfarin remains indispensable in specific high-risk settings, particularly in patients with mechanical heart valves. Importantly, its narrow therapeutic window necessitates regular monitoring and renders anticoagulation control highly susceptible to dietary and pharmacologic influences, underscoring the clinical importance of food–drug interactions. Warfarin functions as a VKA, primarily by interfering with the recycling of vitamin K in the liver. Vitamin K is a crucial component in the synthesis of certain blood clotting factors. The mechanism of warfarin involves inhibiting an enzyme that participates in the recycling process of vitamin K. Vitamin K is essential for the synthesis of various proteins, particularly clotting factors, in the liver. These clotting factors (such as II, VII, IX, and X) play a pivotal role in the blood clotting cascade. Vitamin K is not consumed in the clotting process but is continuously recycled. After being used in the synthesis of clotting factors, vitamin K becomes oxidized. It needs to be converted back to its active form (reduced form) to continue supporting clotting factor synthesis. Warfarin interferes with the vitamin K cycle by inhibiting an enzyme called vitamin K epoxide reductase (VKOR). VKOR is responsible for reducing oxidized vitamin K back to its active form. By inhibiting VKOR, warfarin prevents the recycling of vitamin K. This leads to a reduction in the availability of the active form of vitamin K in the liver. With reduced levels of active vitamin K, the liver’s ability to produce fully functional clotting factors is impaired. This results in the synthesis of less effective clotting factors, affecting the blood clotting process. To ensure that the anticoagulant effect remains within the desired therapeutic range, healthcare providers monitor patients using the INR. The INR provides a standardized measure of blood clotting time. A number of factors may affect INR control in patients taking warfarin. Some factors are inherent, such as genetic variants that contribute to interindividual variability in warfarin dose requirements and difficulty achieving stable anticoagulation. Large Phase III randomized clinical trials have established the efficacy and safety of DOACs compared with VKAs for the prevention and treatment of venous thromboembolism, including pulmonary embolism, and the prevention of cardioembolic stroke in nonvalvular atrial fibrillation (AF), the latter representing the largest share of OAC use worldwide about 65%.^[5]^ For many major indications, due to their favorable practical management and lack of monitoring requirements, DOACs have largely replaced VKAs, except for some high-thrombogenic conditions such as end-stage renal failure and hemodialysis, mechanical heart valves, triple-positive antiphospholipid syndrome), and valvular AF. Vitamin K antagonists remain the reference treatment for patients with mechanical heart valves based on the negative outcome of 1 Phase II randomized clinical trials on dabigatran versus warfarin, which was terminated for major safety drawbacks with dabigatran.^[6]^ With the increasing global use of DOACs, the potential clinical relevance of food–drug interactions warrants continued attention. Nevertheless, VKAs, including warfarin, remain the standard and recommended anticoagulant therapy for patients with mechanical heart valves, as DOACs are contraindicated in this population. Although evidence regarding interactions between persimmon intake and DOACs is currently lacking, further investigation may be warranted as the use of OACs continues to expand. Warfarin exerts its anticoagulant effect primarily by inhibiting the VKOR complex, encoded by the VKORC1 gene, thereby reducing the regeneration of active vitamin K and the synthesis of vitamin K-dependent clotting factors. In addition, the pharmacokinetics of warfarin are largely determined by hepatic metabolism via the cytochrome P450 system, particularly the CYP2C9 isoenzyme, which is responsible for the clearance of the more potent S-enantiomer of warfarin. Genetic polymorphisms in VKORC1 and CYP2C9 are well established contributors to interindividual variability in warfarin dose requirements and anticoagulant sensitivity. Although genetic testing was not performed in the present case, it is plausible that dietary factors could interact with these established pathways and thereby influence anticoagulation control in susceptible individuals. Persimmons contain a variety of bioactive compounds, including vitamin K, tannins, and polysaccharides, which have been reported to affect coagulation-related processes in experimental settings. However, there is currently no direct evidence demonstrating that persimmon constituents alter warfarin metabolism through modulation of CYP2C9 activity or VKORC1 expression. Accordingly, any mechanistic association between persimmon intake and excessive anticoagulation remains speculative and should be interpreted with caution. Moreover, commonly referenced international clinical guidelines, pharmacology textbooks, and drug–food interaction databases do not identify persimmon as a recognized interacting food with warfarin. This absence highlights an important limitation in the existing literature and underscores the need for further clinical and pharmacologic studies to determine whether persimmon-related effects represent a rare idiosyncratic response or an underrecognized dietary interaction in patients receiving warfarin therapy. International clinical guidelines and expert consensus statements consistently emphasize the importance of maintaining dietary consistency and providing structured patient education to minimize food–drug interactions during warfarin therapy. Major guidelines issued by organizations such as the American College of Cardiology/American Heart Association, the European Society of Cardiology, and the American College of Chest Physicians do not identify specific foods, including persimmons, as contraindicated. Instead, these guidelines underscore general management principles, such as avoidance of abrupt dietary changes, regular monitoring of the INR, and ongoing patient education regarding vitamin K-containing foods and other dietary factors that may affect anticoagulation stability. In high-risk populations: particularly patients with mechanical heart valves: these recommendations highlight the need for cautious and individualized anticoagulation management, given the substantial risks associated with both excessive and subtherapeutic anticoagulation. Within this context, the present case suggests that culturally specific and commonly consumed foods, even when not explicitly addressed in international guidelines or drug–food interaction databases, should be considered when unexplained fluctuations in anticoagulation parameters are observed. The harmful results of this trial hampered the development of anti-Xa compounds in this setting. Furthermore, the Prospective Randomized On-X Anticoagulation Clinical Trial Xa (PROACT Xa) trial that was designed to test the efficacy of apixaban versus warfarin in patients with aortic mechanical valves replaced at least 3 months ago was also stopped in September 2022 by the Data and Safety Monitoring Board,^[7]^ but details are currently unpublished. A significant gap exists in the literature regarding large-scale randomized controlled trials directly comparing VKAs and DOACs immediately following bioprosthetic valve replacement. As a result, current recommendations for AF patients suggest considering DOACs with a low level of evidence and class recommendation, starting no sooner than 3 months after the implantation of a bioprosthetic valve.^[8]^ Transcatheter aortic valve implantation patients with no other indications for DOACs should be treated with low-dose aspirin based on current evidence, while for patients with a clear indication for OACs, there is no convincing evidence that DOACs are superior to VKAs.^[9]^

Persimmon (*D kaki*) is originally from the temperate regions of Asia, including South Korea, China, and Japan. With the exception of certain mountainous areas in the central, northern, and some regions of South Korea, it can be cultivated throughout the country. It’s worth noting that the nutritional composition can vary slightly depending on the specific variety of persimmon. Persimmons offer not only delightful flavors but also a wealth of essential nutrients:

1. Vitamins:

Vitamin A: Persimmons are a rich source of β-carotene, a precursor to vitamin A, vital for maintaining healthy skin, vision, and immune function.

Vitamin C: An antioxidant supporting the immune system, promoting skin health, and aiding in the absorption of iron.

2. Dietary fiber:

Persimmons boast a significant amount of dietary fiber crucial for digestive health. Fiber regulates bowel movements, prevents constipation, and supports a healthy gut.

3. Minerals:

Potassium: An electrolyte playing a crucial role in maintaining proper fluid balance, supporting heart health, and regulating blood pressure.

Manganese: Involved in bone formation, blood clotting, and inflammation reduction.

4. Antioxidants:

Persimmons are abundant in various antioxidants, including polyphenols and flavonoids, neutralizing harmful free radicals. Antioxidants contribute to overall health and may reduce the risk of chronic diseases.

5. Calories and macronutrients:

Persimmons are relatively low in calories, making them a healthy snack option. They provide small amounts of natural sugars, such as glucose and fructose, and contain minimal fat and protein.

6. Other nutrients: phytochemicals:

These naturally occurring compounds in plants, found in persimmons, may offer health benefits, enhancing their overall nutritional value.

Within these nutrients, the occurrence of specific elements, such as vitamin K, might be more noticeable in astringent varieties when contrasted with non-astringent ones. Even after reaching maturity, the fruit contains 1% to 2% tannins, requiring a specific processing method like dehulling or persimmon maturation to make it suitable for consumption. Known for its richness in vitamins A and C, this alkaline food is traditionally favored for its various health benefits, such as promoting the secretion of intestinal fluids. The astringent taste in persimmons is attributed to soluble tannins. Tannin compounds are widely present in plant tissues such as fruits, vegetables, and seeds, exhibiting pharmacological effects like astringency and hemostasis. Diospyrin is a tannin compound responsible for the astringent taste in persimmons. However, excessive consumption can potentially result in constipation. This is due to diospyrin causing contraction of the tissues on mucous membrane surfaces within the body when present in significant amounts, leading to a reduction in diarrhea but triggering constipation. Sprinkling alcohol, like soju, on persimmons eliminates the astringent taste. Ethanol is converted to acetaldehyde by enzymes in persimmons, reacting with soluble tannins. There’s an old saying that if you’re gearing up for a “drinking contest,” loading up on non-astringent persimmons beforehand is the way to go. Additionally, they possess the characteristic of binding with proteins or alkaloids. Alongside reported physiological activities such as antibacterial, antioxidant, antitumor effects, and the ability to remove heavy metals, tannins are recognized for their pharmacological properties. Furthermore, persimmons contain abundant functional phenolic compounds, such as catechin, epicatechin, epicatechingallate, epigallocatechin, epigallocatechingallate, and betulinic acid, which are commonly found in green tea. These substances are known for their antioxidant properties, antiaging effects, prevention of cardiovascular diseases, and anticancer effects.

The interaction between warfarin and persimmons is a notable consideration for individuals taking warfarin. Warfarin is an anticoagulant that works by inhibiting the synthesis of certain blood clotting factors, therefore it is crucial for patients on warfarin to maintain a stable level of anticoagulation, as both excessive anticoagulation and insufficient anticoagulation can lead to serious health risks. In 2004, Asgar et al^[10]^ demonstrated that polysaccharides derived from persimmon fruits constitute a group of heteropolysaccharides with an average molecular weight of 1.3 × 10^5^ Da. These polysaccharides are primarily composed of arabinose, mannose, rhamnose, galactose, and glucose. Building upon their findings, Zhang et al^[11]^ revealed that the sulfated polysaccharide extracted from persimmon shows promising anticoagulant properties, serving as a potential alternative to antithrombotic compounds. The anticoagulant activity of sulfated persimmon polysaccharide was observed to inhibit the intrinsic coagulant process and thrombin-mediated fibrin formation; however, it did not affect the extrinsic coagulant process. Persimmons, particularly certain varieties such as the Japanese persimmon (*D kaki*), contain vitamin K. Vitamin K is essential for blood clotting, and its intake can potentially affect the anticoagulant activity of warfarin. This is because warfarin works by antagonizing vitamin K, so changes in vitamin K intake can impact the drug’s effectiveness. In Korea, persimmons are classified into 3 main types based on their ripeness and preparation: “Dangam,” enjoyed when firm and unripe; fully ripened “Hongsi” (also referred to as “Mulleong-gam” in cultural language); and “Gotgam” dried before consumption. Dried persimmons, particularly after the removal of astringency, have been a prominent fruit-drying product in South Korea for an extended period. More than 50% of astringent persimmons are transformed into dried persimmons, known as “gotgam” in Korea. To the best of the authors’ knowledge, there have been no reports documenting persimmon-induced excessive anticoagulation in patients receiving warfarin therapy. It is postulated that the effect on the function of VKAs may not solely arise from vitamin K intake through persimmons but may also involve the diverse array of nutritional elements inherent to persimmons. However, it is recognized that certain foods, particularly those abundant in vitamin K, have the potential to disrupt the anticoagulant effects of warfarin. One such example is fully ripened persimmon, known as “Hongsi,” and dried persimmon, referred to as “gotgam” in Korean, which are traditional dried fruit snacks popular in East Asia. Typically prepared during the winter months, this delicacy is created by air-drying Oriental persimmons. The process involves peeling and drying the persimmons, followed by tying and hanging them in a sunny, well-ventilated area, such as under the eaves of a house. In South Korea, at least 3 varieties of persimmons (Dangam, Hongsi, Gotgam, etc) are commonly enjoyed, each boasting unique textures and nutritional profiles. It is understood that these diverse forms of Korean persimmons may warrant further research and consideration regarding potential interactions with warfarin and other medications. Furthermore, it should be noted that an approach limited to persimmon-oriented vitamin K alone is insufficient. A thorough examination of the concomitant elevation in PT and aPTT is warranted. This underscores the importance of considering factors affecting both the intrinsic and extrinsic pathways, as well as the common coagulation pathway, as components of persimmons may influence them.

An intriguing aspect of warfarin administration is the considerable variability in the dosages prescribed across different countries. In Korea, healthcare professionals are limited to prescribing warfarin in doses of 2 or 5 mg, whereas the United States utilizes a more diverse spectrum of dosages, including 1 mg/2 mg/2.5 mg/3 mg/4 mg/5 mg/6 mg/7.5 mg/10 mg, each represented by distinct colors such as pink, lavender, green, brown, blue, peaches, teal, yellow, and white. In many countries, including the United States, warfarin tablets are color-coded to indicate their strength, but in Japan and South Korea, warfarin tablets may not have color-coded formulations. Medical practitioners frequently encounter patients in the emergency department or outpatient clinics who are anticoagulated with warfarin but are unaware of their dosage. To prevent complications such as hypercoagulation due to warfarin overdose, manufacturers of both brand-name and generic warfarin in the United States have standardized the color of each strength. Although the shape and shade may vary, the colors of warfarin remain consistent. Additionally, the shape and shade of the tablets may vary, but the color-coding system remains consistent to help patients and healthcare providers easily identify the dosage of warfarin being taken. Consistency in warfarin colors across all brands and manufacturers is crucial given its status as a high-risk medication requiring frequent INR monitoring for safety and efficacy. It is imperative for both patients and providers to be aware of the exact warfarin dosage being taken at home. Simply memorizing these colors can enhance the quality of patient counseling. To aid in remembering the colors, a mnemonic commonly used in the USA is: “Please Let Granny Brown Bring Peaches To Your Wedding.” Alternatively, Australia provides an emergency medicine-related mnemonic: “Probably Leaves Granny Bleeding Bloody Profusely, Thank You Warfarin.” One consideration is that, regrettably, in South Korea, the available dosages of warfarin for prescription are limited to 2 or 5 mg. To adjust the dosage, it is most often recommended to split the tablets. This is in contrast to the United States, where a variety of dosages can be prescribed (1, 2, 2.5, 3, 4, 5, 6, 7.5, and 10 mg), allowing for more flexible dosing options. Consequently, in Korea, prescription options for warfarin dosages are severely restricted.

To the best of the authors’ knowledge, there have been no reports documenting persimmon-induced excessive anticoagulation in patients receiving warfarin therapy. It is hypothesized that the impact on the function of VKAs may not solely stem from vitamin K intake through persimmons but may also involve the diverse array of nutritional elements present in persimmons. However, it is recognized that certain foods, particularly those abundant in vitamin K, can potentially counteract the anticoagulant effects of warfarin.

The patient expressed surprise that a commonly consumed fruit could interfere with anticoagulation therapy, stating, “I was surprised to learn that eating persimmons could affect my blood thinner medication… I’m grateful that the issue was found early during a routine checkup.” This perspective highlights the importance of dietary counseling in patients receiving VKAs. Internationally, food–drug interactions involving warfarin have been reported most frequently in association with dietary components rich in vitamin K, herbal supplements, and selected fruits and vegetables containing bioactive compounds that may influence coagulation pathways or warfarin metabolism. Previous reports have described fluctuations in anticoagulation control related to foods such as leafy green vegetables, cranberry products, mango, grapefruit, and various herbal preparations; however, these observations are largely anecdotal and heterogeneous with respect to patient characteristics, dietary exposure, and clinical outcomes. Notably, persimmon has not been systematically evaluated or highlighted in major international reviews, clinical guidelines, or commonly used drug–food interaction databases. Within this context, the present case expands the spectrum of potential dietary factors associated with excessive anticoagulation in patients receiving warfarin therapy. Although the global prevalence and clinical significance of persimmon-related interactions remain unknown, this observation reinforces a broader clinical principle: unexpected fluctuations in anticoagulation parameters may arise from culturally specific and commonly consumed foods that are not explicitly addressed in international recommendations. These findings underscore the importance of comprehensive dietary assessment and individualized patient education as integral components of warfarin management, particularly in high-risk populations requiring lifelong anticoagulation.

From a practical clinical perspective, this case highlights the importance of comprehensive dietary assessment and consistent patient education in the management of warfarin therapy, particularly in high-risk populations such as patients with mechanical heart valves. Rather than focusing exclusively on well-recognized vitamin K-rich foods, clinicians should emphasize the broader principle of dietary consistency and maintain vigilance for abrupt changes in the consumption of culturally specific or seasonal foods. Structured education delivered by physicians, pharmacists, and other healthcare professionals plays a central role in identifying potential food–drug interactions, including those related to over-the-counter supplements and traditional dietary products. Furthermore, closer monitoring of the INR should be considered when unexpected dietary changes are identified, even in the absence of foods explicitly listed in clinical guidelines. Collectively, these practical measures may help reduce the risk of excessive anticoagulation and enhance the overall safety of long-term warfarin therapy. The unique clinical value of this case lies in highlighting a clinically meaningful food–drug interaction involving a culturally specific and commonly consumed food in a high-risk patient requiring lifelong warfarin therapy, thereby emphasizing the need for culturally tailored dietary assessment and heightened clinical vigilance when unexplained INR fluctuations occur, while also underscoring the limitations inherent to a single-case observation: most notably the inability to establish causality, the absence of pharmacogenetic evaluation of CYP2C9 and VKORC1, and the lack of biochemical quantification of persimmon intake: which collectively point to the need for future larger-scale observational, pharmacokinetic, and pharmacogenetic studies to determine whether such interactions represent rare idiosyncratic events or underrecognized contributors to anticoagulation instability.

## 4. Conclusions

This clinical report represents the first documented instance of a hypercoagulable state possibly triggered by the commonly consumed persimmon in South Korea. Despite leveraging the authors’ expertise and thorough investigation, fully understanding persimmon-induced hypercoagulability presents a challenge due to the scarcity of clinical reports and research, especially regarding the underlying mechanism and clinical progression. However, ongoing efforts in clinical reporting and research are expected to shed light on this phenomenon in future studies. Additionally, multicenter, multinational studies investigating VKAs like warfarin in South Korea are considered crucial.

## Author contributions

**Conceptualization:** JunSeok Kim, SongAm Lee, HyeongJu Moon, WooSurng Lee.

**Data curation:** WooSurng Lee.

**Formal analysis:** WooSurng Lee.

**Investigation:** WooSurng Lee.

**Methodology:** WooSurng Lee.

**Software:** WooSurng Lee.

**Writing – original draft:** WooSurng Lee.

**Writing – review & editing:** WooSurng Lee.
